# Using EMRALD to assess baseline body mass index among children living within and outside communities participating in the Ontario, Canada Healthy Kids Community Challenge

**DOI:** 10.1371/journal.pone.0213443

**Published:** 2019-04-11

**Authors:** Sarah K. Orr, Karen Tu, Sarah Carsley, Hannah Chung, Laura Holder, Shirin Jabbari, Daniel W. Harrington, Heather Manson

**Affiliations:** 1 Department of Health Promotion, Chronic Disease, and Injury Prevention, Public Health Ontario, Toronto, Ontario, Canada; 2 Department of Family and Community Medicine, Faculty of Medicine, University of Toronto, Toronto, Ontario, Canada; 3 Toronto Western Hospital Family Health Team, University Health Network, Toronto, Ontario, Canada; 4 ICES, Toronto, Ontario, Canada; 5 School of Public Health and Health Systems, University of Waterloo, Waterloo, Ontario, Canada; 6 Clinical Public Health Division, Dalla Lana School of Public Health, University of Toronto, Toronto, Ontario, Canada; University of Auckland, NEW ZEALAND

## Abstract

**Objectives:**

The Healthy Kids Community Challenge is a large-scale, centrally-coordinated, community-based intervention in Ontario, Canada that promotes healthy behaviours towards improving healthy weights among children. With the goal of exploring tools available to evaluators, we leveraged electronic medical records from primary care physicians to assess child weights prior to launch of the Healthy Kids Community Challenge. This study compares the baseline (i.e. pre-intervention) prevalence of overweight and obesity in children 1–12 years of age living within and outside Healthy Kids Community Challenge communities.

**Design:**

Cross-sectional analysis of a primary care patient cohort.

**Setting:**

Electronic Medical Record Administrative data Linked Database (EMRALD) in Ontario, Canada.

**Participants:**

A cohort of 19 920 Ontario children who are rostered to an EMRALD physician. Children were 1–12 years of age at a primary care visit with recorded measured height and weight, between January 1, 2014 and December 31, 2015.

**Outcome measure:**

Overweight and obesity as determined by age- and sex-standardized body mass index using World Health Organization’s Growth Standards.

**Results:**

In Healthy Kids Community Challenge communities, 25.6% (95% CI 24.6–26.6%) of children had zBMI above normal (i.e. >1) compared to 26.7% (95% CI 25.9–27.5%) for children living outside of Healthy Kids Community Challenge communities.

**Conclusions:**

Despite some differences in sociodemographic characteristics, zBMI of children aged 1–12 years were similar inside and outside of Healthy Kids Community Challenge community boundaries prior to program launch.

## Introduction

Public health policy-makers and practitioners are increasingly expected to demonstrate the impacts of their programs on population health outcomes.[1] However, assessing the impacts of large-scale programs intended to improve the health of the population can be challenging.[2,3] The publically-funded nature of such programs means that they are often working within constrained resources, with tension between funding the program and its evaluation. Evaluators must remain nimble and seek timely and feasible solutions to maintain rigour in order to meet the public health policy-makers’ and practitioners’ needs. With the goal of exploring tools available to evaluators, we used electronic medical records from primary care physicians to assess child weights prior to the start of the Healthy Kids Community Challenge (HKCC).

The HKCC is a large-scale, centrally-coordinated, community-based intervention in Ontario, Canada that promotes healthy behaviours towards improving healthy weights among children.[4] Designed by Ontario’s Ministry of Health and Long-Term Care (MOHLTC) based on the Ensemble Prévenons l’Obésité des Enfants’ (EPODE) model, the HKCC was launched in September 2015 in 45 selected communities. MOHLTC provided the communities with coordination, funding, training, and other resources (e.g., social marketing messages and tools) to implement community programs and activities that address locally-identified needs. Programs and activities implemented by communities varied widely. Some examples include the installation of water-filtration systems in schools (i.e., to promote water and displace sugar-sweetened beverage consumption) and offering subsidized physical activity programming or equipment. Six of the communities were funded and implemented through Aboriginal Community Health Access Centres or Community Health Centres (AHAC/CHC) as part of an “Aboriginal Stream”, while the remaining 39 communities were municipally-based. The 39 municipal communities, which are the focus of the current analysis, had programs and activities running from early 2016 to September 2018. The impacts of the HKCC in Aboriginal Stream communities are being evaluated under a different component of Public Health Ontario’s evaluation of the HKCC. [5]

Primary data collection of direct body weight measures can be challenging for the evaluation of large-scale interventions because it requires sufficient time for planning, multi-stakeholder involvement, and significant resources. Using survey data to estimate healthy weights in Ontario is also challenging, particularly for children.[6] Although the Canadian Health Measures Survey (CHMS) and the Canadian Community Health Survey (CCHS)-Nutrition survey collect directly measured anthropometric data, both surveys have sample sizes that restrict sub-provincial estimates. Further, while CHMS has biennial collection cycles, it does not survey children younger than 3 years of age; and while CCHS-Nutrition measures heights and weights of children as young as 2 years of age, it has only been administered in 2004 and 2015.

Given the limitations of survey data and the challenges involved with primary data collection, we chose to leverage secondary data sources that store measured heights and weights. The Electronic Medical Record Administrative data Linked Database (EMRALD) contains patient electronic medical records (EMRs) from participating primary care physicians across Ontario. This data includes routinely-collected, objectively measured heights and weights.[7]. In Canada and Ontario, the standard of care is to routinely collect height and weight at well-baby/child visits, which are recommended at 1 week, 2, 4, 6, 9, 12 and 18 months, each year from 2–5 years and then every 1–2 years until 18 years of age.[8] One study reported Ontario children have a median of 19 primary healthcare visits in the first two years of life, and includes at least 7 well-child visits as a result of the immunization schedule.[9] Two studies have shown for children who attend well-baby/child visits, between 84.7% and 89.9% of visits had a height and weight recorded on the same date.[6,10] These studies assessed the practicality of using EMRALD data for obesity surveillance in children and suggest it is feasible to use EMRALD for evaluations of population-level obesity interventions like the HKCC.[6,10]

Our primary objective was to assess the pre-intervention difference in the prevalence of high body mass index z-scores (zBMI) among children 1–12 years living in HKCC communities, compared to those living outside of HKCC communities, using EMRALD data. We also examined the association between sociodemographic and health characteristics and zBMI in our study cohort, as well as the generalizability of our study cohort with all children in Ontario.

## Methods

### Data

EMRALD contains data collected from over 2% of family physicians in Ontario who contribute their patients’ electronic medical record (EMR) data.[7] Physician participation is voluntary and there is no monetary incentive. Characteristics of contributing physicians and their patients have been detailed previously.[11] At the time of this analysis, EMRALD included 310 physicians from 41 clinics. Height and weight measurements performed during primary care visits were extracted for all children. Health administrative databases included but were not limited to the Ontario Registered Persons Database (RPDB), which has demographic information for individuals with an Ontario health card number, and the Ontario Health Insurance Plan (OHIP) database, which contains physician billing records for all billing claims. Ontario has a single-payer healthcare system, thus virtually all Ontario residents have a health card number and are represented in the RPDB. These datasets were linked using unique encoded identifiers and analyzed at ICES. All data linkages and analyses were performed by ICES personnel, with input from PHO.

Data on HKCC community postal codes was derived from a community boundary mapping process. Community-defined boundaries for the 39 participating municipal communities were converted to census dissemination areas within a geographic information system, using the 2013 version of the Postal Code Conversion File from Statistics Canada.[12] This postal code data was linked to children’s postal codes in EMRALD to define those children living within and outside of HKCC communities.

The six Aboriginal Stream communities were not included in this analysis, because they are being evaluated under a different component of Public Health Ontario’s (PHO) evaluation.[5] The likelihood of an HKCC Aboriginal Stream participant being included in EMRALD is negligible because the primary care setting for children who participate in the Aboriginal Stream communities’ initiatives is likely to be an AHAC/CHC, and not an EMRALD physician’s office.

### Study cohort

We established a cohort of children who were residents of Ontario, rostered to an EMRALD physician, with postal codes within our list of HKCC and non-HKCC postal codes, alive at the time of cohort creation, with congruent demographic information in EMRALD and the RPDB, for whom we could link their EMRALD identifier to administrative data, and with valid zBMI measurements (see below) within the study period of January 1, 2014 and December 31, 2015. Following exclusions, the cohort was split into HKCC and non-HKCC communities. Our final analytic cohort consisted of 19 920 children.

To facilitate generalizability assessments, we used the RPDB to create a comparison cohort of all children in Ontario. We used the same inclusion criteria as for the study cohort (i.e. alive and ages 1–12 as of January 1, 2015), and assigned July 1, 2015 as the index date to measure all baseline characteristics. Our Ontario population cohort consisted of 1,760,946 children.

### Exposure variable

Children living within the 39 defined municipal HKCC community boundaries were presumed to be exposed to HKCC interventions. Children living in communities outside these boundaries were assumed to not have been exposed to HKCC interventions.

### Outcome variables

We categorized each child in the cohort into weight status categories using WHO recommended zBMI cut-offs, for example a zBMI >-2 to ≤1 is “normal”.[13,14] WHO cut-offs approximate percentiles and coincide with growth terminology categories determined by age. Since the age range in our cohort includes the age when a change in terminology occurs (5 years), for consistency, we categorized the outcome using zBMI cut-off categories.

Measured height and weight on the same date were used to calculate the child’s body mass index (BMI), which was then age and sex-standardized using the World Health Organization’s Growth Standards and Reference Charts.[13] Valid zBMIs were defined as those where height, weight, and calculated zBMI score were within a plausible range. We defined biologically implausible values (BIV) based on a modified set of validated rules, specifically where recorded weight measurements were <1 kg or >300 kg, height measurements were <20 cm or >221 cm, and where zBMI scores were <-5 or >5.[15,16]

If a child had more than one valid zBMI measurement in the observation period, we selected the measurement from the most recent well-baby/child visit since it has been shown that rates of overweight and obese status were higher when measurements were taken from other visit types.[6] If the child did not have a zBMI measurement from a well-baby/child visit, we selected their zBMI value from their most recent visit with unknown visit type or their most recent ‘sick’ visit if the child only had ‘sick’ visits during the entire observation period, as defined by OHIP billing claims on the same date.

### Baseline characteristics

Child’s sex, age, and sociodemographic information on their visit date were determined using the RPDB. Neighbourhood income quintile and rural residency status (i.e., living in a community with size <10 000 people) were determined by linking each child’s postal code to 2006 Census information.[17]

Immigration status was determined by linking each child’s unique encrypted identifier to the Immigration, Refugees and Citizenship Canada’s Permanent Resident Database. For those children without landing dates, we then sought to determine whether their birth mother was a landed immigrant; we identified their birth mothers using the Mother-Baby Linked Database, which links the child’s hospitalization record at birth with their mother’s delivery hospitalization record only for deliveries that occurred in Ontario. We subsequently linked the mother’s unique encrypted identifier with IRCC. Children were categorized as being part of an immigrant household if they or their birth mother were identified as immigrants. Ethnicity is not routinely collected in administrative databases in Ontario. However, South Asian and Chinese ethnicity were determined using a validated algorithm based on surname from the RPDB.[18]

The presence of medical comorbidities were identified using diagnostic, procedure, and fee codes for a history of asthma, diabetes (types 1 and 2), inflammatory bowel disease, complex chronic conditions/congenital disorders, and mental health conditions.[19–23]

We measured each child’s illness burden in the year prior to their visit date using The Johns Hopkins ACG System Version 7, which has been used previously in the Canadian health care context.[24] ACG System Resource Utilization Bands were used to represent groups of individuals with comparable expected resource use (from a non-user to a high user).[25]

Due to the administrative nature of the data used and the exclusion of children missing zBMI data, there were no missing data in the analytic cohort, with the exception of a small number of children for whom neighbourhood income quintile could not be determined.

### Statistical analyses

To assess generalizability of the EMRALD cohort, we compared the baseline characteristics of the EMRALD cohort and all children in Ontario using chi-square test statistics and calculated standardized differences.[26] To compare the proportion of children in each zBMI cut-off category between children living within HKCC communities versus those living outside HKCC community boundaries, we used chi-square test statistics and calculated 95% exact binomial confidence limits. We further performed these zBMI cut-off category comparisons stratified by sex. In addition, we examined the proportion of children who would be classified as obese or severely obese (zBMI>3) dependent on visit type when zBMI was measured.

To assess the associations between selected baseline characteristics and zBMI, we conducted linear regression analyses were zBMI was modeled as a continuous outcome. For these linear regression analyses, we excluded children who were classified as underweight (~2%) in order to improve model fit and determine the association between selected characteristics and zBMI ranging from ‘normal’ to ‘overweight’ and ‘obese’. We did not include inflammatory bowel disease in the models because of its low prevalence in our cohort, which would consequently cause model convergence issues.

This study was approved by the ethics review board at Public Health Ontario and the Research Ethics Board at Sunnybrook Health Sciences Centre, Toronto, Canada.

## Results

Table 1 shows the baseline characteristics of children in the cohort by community type, specifically HKCC (n = 7 382) and non-HKCC (n = 12 538). Overall, baseline characteristics show that these are two relatively comparable populations. However, there were a few notable differences between children living in HKCC communities versus outside of HKCC communities. First, there was a higher proportion of children in the youngest age group living in HKCC communities (36.0% 1–3 y vs. 32.9% in non-HKCC communities); in contrast to a higher proportion of children in the oldest age groups in non-HKCC communities (22.7% 9–12 y vs. 18.5% in HKCC communities). Second, HKCC communities in the cohort had a higher proportion of children living in rural communities (19.3% vs 15.8% in non-HKCC communities). Third, there was a higher proportion of children in the lowest neighbourhood income quintile living in HKCC communities (18.5% vs. 10.1% in non-HKCC communities). Fourth, HKCC communities in the cohort had a higher proportion of children living in immigrant households (14.8% vs. 11.1% in non-HKCC communities). Finally, there was a lower proportion of children in HKCC communities with asthma (8.8% vs. 10.1% in non-HKCC communities) or a mental health condition (17.9% vs. 20.6% in non-HKCC communities), however, there was a higher proportion of children with a complex chronic disease or congenital disorder (7.2% vs. 6.4%).

**Table 1:** Comparison of baseline characteristics between children 1–12 living in HKCC communities vs. non-HKCC communities (N = 19 920).

	HKCC (N = 7 382)		Non-HKCC (N = 12 538)			
	n	%	n	%	Std Diff	P-value*
*Sex*						
Female	3 546	48.0	6 088	48.6	0.010	0.478
Male	3 836	52.0	6 450	51.4		
*Age group*						
1–3	2 658	36.0	4 131	32.9	0.109	< .001
4–8	3 361	45.5	5 556	44.3		
9–12	1 363	18.5	2 851	22.7		
*Neighbourhood Income Quintile*						
1- Lowest	1 364	18.5	1 266	10.1	0.247	< .001
2	1 258	17.0	2 508	20.0		
3	1 570	21.3	2 696	21.5		
4	1 616	21.9	3 122	24.9		
5- Highest	1 557	21.1	2 911	23.2		
Missing income quintile	17	0.2	35	0.3		
*Rural Community*						
Yes	1 427	19.3	1 982	15.8	0.093	< .001
*Immigration Status*						
Child—Yes	106	1.4	120	1.0	0.116	< .001
Child—No but Mother is an immigrant	991	13.4	1 269	10.1		
Child—No and Mother is not an immigrant	5 538	75.0	9 731	77.6		
Child—No but Mother’s immigration status is unknown	747	10.1	1 418	11.3		
*Ethnicity*						
Chinese	155	2.1	356	2.8	0.055	0.001
South Asian	122	1.7	165	1.3		
Other ethnicities	7 105	96.2	12 017	95.8		
*History of medical co-morbidities*						
Asthma	651	8.8	1 261	10.1	-0.042	0.004
Diabetes	14	0.2	12	0.1	0.025	0.076
Inflammatory bowel disease	0–5^†^	0.00–0.07	0–5^†^	0.00–0.04	-0.018	0.278
Complex chronic condition / congenital disorder	532	7.2	803	6.4	0.032	0.029
Any mental health condition	1 322	17.9	2 584	20.6	-0.069	< .001
*Resource Utilization Bands*						
0 (non-user)	565	7.7	1 029	8.2	0.076	< .001
1 (low)	1 503	20.4	2 432	19.4		
2	3 631	49.2	5 929	47.3		
3	1 507	20.4	2 882	23.0		
4	162	2.2	257	2.0		
5 (high)	14	0.2	9	0.1		

* P-values are from comparisons between children living in HKCC and non-HKCC communities.

^†^ Cells suppressed due to small counts (<6).

There was no difference in the proportion of children in each zBMI category when comparing HKCC and non-HKCC communities (Table 2). Overall, 71.7% of children had a normal zBMI as defined by WHO (>-2 to ≤1). In HKCC communities, 25.6% (95% CI 24.6–26.6%) of children had zBMI above normal (i.e. >1) compared to 26.7% (95% CI 25.9–27.5%) of children in non-HKCC communities. When broken down into sex categories, there was only one statistical difference between HKCC and non-HKCC communities (S1 Table). Specifically, there was a lower proportion of females in HKCC communities with zBMI >2 to ≤3 (5.4%, 95% CI 4.7–6.2%) compared to non-HKCC communities (6.5%, 95% CI 5.9–7.1%). When broken down into age groups, in HKCC communities there was a higher proportion of children 1–3 years old with zBMI classified as ‘normal’ (72.6%, 95% CI 70.8–74.3%), specifically a zBMI of >-2 to ≤1, compared to non-HKCC communities (70.4%, 95% CI 68.9–71.7%) (S2 Table). There was a corresponding lower proportion of children 1–3 years old with a zBMI classified as ‘overweight’ (>2 to ≤3) in HKCC communities (5.0%, 95% CI 4.2–5.9%) compared to non-HKCC communities (6.3%, 95% CI 5.5–7.0%). In age groups 4–8 and 9–12 years old there were no differences between HKCC and non-HKCC communities (S2 Table).

**Table 2:** Proportion of children in body mass index z-score (zBMI) categories overall and by HKCC community status, ages 1–12 (N = 19 920).

	HKCC Communities		Non-HKCC Communities			Total	
	N	% (95% CI)	N	% (95% CI)	p-value	N	% (95% CI)
≤-2	152	2.1 (1.8–2.4)	243	1.9 (1.7–2.2)	0.56	395	2.0 (1.8–2.2)
>-2 to ≤1	5 342	72.4 (71.3–73.4)	8 946	71.4 (70.6–72.1)	0.13	14 288	71.7 (71.1–72.4)
>1 to ≤2	1 284	17.4 (16.5–18.5)	2 255	18.0 (17.3–18.7)	0.29	3 539	17.8 (17.2–18.3)
>2 to ≤3	466	6.3 (5.8–6.9)	852	6.8 (6.4–7.3)	0.19	1 318	6.6 (6.3–7.0)
>3	138	1.9 (1.6–2.2)	242	1.9 (1.7–2.2)	0.76	380	1.9 (1.7–2.1)

To assess any information bias that might arise based on the type of visit where zBMI measurements were taken, visit type was analyzed by community type. Among children from HKCC communities, most measurements were performed at well-baby/child visits as opposed to ‘sick’ visits (70.0%; 95% CI 69.0–71.1% vs. 23.8%; 95% CI 23.1–24.6%); this was similar in non-HKCC communities (67.5%; 95% CI 66.7–68.3% vs. 20.6%; 95% CI 19.7–21.5%). There was a higher proportion of zBMI measured at well-baby/child visits (p<0.001) and a corresponding lower proportion of zBMI measured at ‘sick’ visits (p<0.001) in HKCC communities compared to non-HKCC communities. This difference was likely due to the higher proportion of young children in HKCC communities (Table 1) because younger children have more well-baby/child visits.[6] The prevalence of zBMI greater than 2, corresponding to overweight and obesity among 0–4 year olds, and obesity and severe obesity among 5–19 year olds, was 7.0% using measurements performed at well-baby/child visits only (n = 13 630); 7.3% when using measurements performed at well-baby/child and unknown visit types (n = 15 417), and 8.6% when using measurements performed at well-baby/child, unknown, and ‘sick’ visits (n = 19 920). Despite the higher proportion of well-baby/child zBMI measurements for children in HKCC communities, the current results show that the proportion of children in each zBMI cut-off category was similar between HKCC and non-HKCC communities.

Overall in the cohort in the fully adjusted model, higher zBMI was associated with older age, living in neighbourhoods in the lowest income quintile (relative to the highest), having a history of asthma or diabetes, and being in higher resource utilization bands (relative to non-users) (Table 3); whereas lower zBMI was associated with being of female sex, being of Chinese or South Asian ethnicity (relative to all other ethnicities), and having complex chronic conditions or a congenital disorder. There was a trend in the overall cohort towards lower zBMI and being from an immigrant household, however, this only reached significance among children from HKCC communities. When stratified by community type, the directions of associations were largely consistent with the overall cohort (Table 3); however, certain relationships were no longer significant, likely due to the lower sample size within strata.

**Table 3:** Association between baseline characteristics and zBMI, overall and by HKCC community status, adjusted (ages 1–12, with zBMI >-2, N = 19 474)*.

	Overall n = 19 474	HKCC Communities n = 7 213	Non-HKCC Communities n = 12 261
	Estimate (95% CI)	Estimate (95% CI)	Estimate (95% CI)
***Sex***			
Female	-0.10 (-0.13,-0.07)^§^	-0.11 (-0.17,-0.06)^§^	-0.09 (-0.13,-0.05)^§^
Male	REF	REF	REF
***Age in months (continuous)***	0.02 (0.01,0.02)^§^	0.03 (0.02,0.03)^§^	0.01 (0.00,0.02)^§^
***Income quintile***			
1- Lowest	0.15 (0.10,0.21)^§^	0.11 (0.02,0.19)^†^	0.19 (0.12,0.27)^§^
2	0.04 (-0.01,0.09)	0.01 (-0.07,0.10)	0.06 (0.00,0.12)
3	0.04 (-0.01,0.09)	-0.02 (-0.10,0.06)	0.08 (0.02,0.14)^†^
4	0.02 (-0.02,0.07)	-0.02 (-0.10,0.06)	0.04 (-0.01,0.10)
5- Highest	REF	REF	REF
***Rural***	0.03 (-0.01,0.07)	0.01 (-0.06,0.07)	0.04 (-0.01,0.10)
***Immigrant***			
Immigrant household	-0.04 (-0.09,0.01)	-0.08 (-0.15,0.00)^†^	-0.01 (-0.07,0.06)
Non-immigrant household	REF	REF	REF
***Ethnicity***			
Chinese	-0.21 (-0.31,-0.11)^§^	-0.16 (-0.34,0.02)	-0.24 (-0.36,-0.12)^§^
South Asian	-0.33 (-0.46,-0.19)^§^	-0.36 (-0.57,-0.16)^§^	-0.30 (-0.48,-0.12)^‡^
Other ethnicities	REF	REF	REF
***Chronic conditions***			
Asthma	0.20 (0.14,0.25)^§^	0.17 (0.07,0.26)^§^	0.22 (0.15,0.28)^§^
Diabetes	0.65 (0.22,1.08)^‡^	0.76 (0.17,1.34)^†^	0.50 (-0.13,1.14)
Complex chronic condition / congenital disorder	-0.07 (-0.14,-0.01)^†^	-0.12 (-0.22,-0.02)^†^	-0.04 (-0.12,0.04)
Any mental health diagnosis	0.00 (-0.05,0.04)	-0.03 (-0.10,0.05)	0.01 (-0.05,0.06)
***Resource Utilization Bands***			
0 (non-user)	REF	REF	REF
1–2 (low)	0.06 (0.00,0.12)	0.05 (-0.05,0.15)	0.06 (-0.02,0.13)
3	0.10 (0.03,0.16)^‡^	0.12 (0.01,0.23)^†^	0.08 (0.00,0.16)
4–5 (high)	0.11 (-0.02,0.23)	0.06 (-0.13,0.26)	0.13 (-0.02,0.29)

* Linear regression models (overall, HKCC communities, and non-HKCC communities) were adjusted for all variables included in table.

^†^<p<0.05;

^‡^ p<0.01;

^§^p<0.001

Finally, baseline characteristics of the EMRALD cohort (n = 19 920) were compared to the general population of children in Ontario aged 1–12 years (n = 1 760 946) (Table 4). Generally, differences between the EMRALD cohort and all Ontario children were greater than the differences by community type within the EMRALD cohort (Table 1). Compared to all children in Ontario, the EMRALD cohort is significantly younger, living in higher income neighbourhoods, more rural, with fewer immigrant households, fewer children of Chinese or South Asian ethnicity, with lower prevalence of asthma and mental health conditions, and with higher users of health care.

**Table 4:** Comparison of baseline characteristics between children in the EMRALD cohort and all Ontario children, 1–12 years old.

	Ontario Cohort (N = 1,760,946)		EMRALD cohort (N = 19,920)			
	n	%	n	%	Std Diff	P-value*
*Sex*						
Female	857 653	48.7	9 634	48.4	0.007	0.339
Male	903 293	51.3	10 286	51.6		
*Age group*						
1–3	362 514	20.6	6 789	34.1	0.381	< .001
4–8	771 702	43.8	8 917	44.8		
9–12	626 730	35.6	4 214	21.2		
*Neighbourhood Income Quintile*						
1- Lowest	343 899	19.5	2 630	13.2	0.176	< .001
2	321 717	18.3	3 766	18.9		
3	351 324	20.0	4 266	21.4		
4	391 615	22.2	4 738	23.8		
5- Highest	344 992	19.6	4 468	22.4		
Missing income quintile	7 399	0.4	52	0.3		
*Rural Community*						
Yes	185 317	10.5	3 409	17.1	0.192	< .001
*Immigration Status*						
Child—Yes	61 816	3.5	226	1.1	0.364	< .001
Child—No but Mother is an immigrant	379 174	21.5	2 260	11.3		
Child—No and Mother is not an immigrant	1 078 350	61.2	15 269	76.7		
Child—No but Mother’s immigration status is unknown	241 606	13.7	2 165	10.9		
*Ethnicity*						
Chinese	85 224	4.8	511	2.6	0.213	< .001
South Asian	75 020	4.3	287	1.4		
Other ethnicities	1 600 702	90.9	19 122	96.0		
*History of medical co-morbidities*						
Asthma	259 510	14.7	1 912	9.6	-0.158	< .001
Diabetes	3 593	0.2	26	0.1	-0.018	0.022
Inflammatory bowel disease	448	0.0	0–5^†^	0.00–0.03	-0.012	0.174
Complex chronic condition / congenital disorder	118 798	6.7	1 335	6.7	-0.002	0.804
Any mental health condition	408 162	23.2	3 906	19.6	-0.087	< .001
*Resource Utilization Bands*						
0 (non-user)	400 484	22.7	1 594	8.0	0.420	< .001
1 (low)	295 458	16.8	3 935	19.8		
2	679 081	38.6	9 560	48.0		
3	351 688	20.0	4 389	22.0		
4	31 891	1.8	419	2.1		
5 (high)	2 344	0.1	23	0.1		

* P-values are from comparisons between children in the EMRALD cohort and all Ontario children.

^†^ Cells suppressed due to small counts (<6).

## Discussion

This study demonstrates the utility of electronic medical records for the baseline assessment of a large-scale provincial intervention when it is not feasible to collect primary data, and existing surveys do not adequately cover the outcome (directly measured BMI), population (children aged 1–12 years), or level of exposure (community-based) over the time period of the program. Our study shows that baseline BMI z-scores were similar between children living within and outside of HKCC intervention communities, despite some differences in sociodemographic characteristics. Further, the prevalence of overweight and obesity in the cohort overall was similar to national estimates from surveys using objective measures, which is 27.0% for 3–19 year olds.[27] Together, these findings suggest that prior to the program launch, the weight status of children living in HKCC intervention communities was comparable to Ontario children living outside of HKCC communities.

Due to the data linkage between anthropometric measurements and health administrative databases at the individual level, we were able to both characterize children living in HKCC and non-HKCC communities and determine factors associated with high zBMI values. Regarding the former, within our study cohort, children living in HKCC communities were broadly similar to those living in non-HKCC communities. However, there was a statistically significant trend among children in HKCC communities towards being younger, from lower income neighbourhoods, living rurally, being from an immigrant household, being not of Chinese or South Asian ethnicity, and having less medical co-morbidity compared to children in non-HKCC communities. We found that higher zBMI values were associated with being older (vs. younger) and male (vs. female), and lower-income (vs. higher) which are consistent with Canadian surveys such as the CHMS.[27–30] Higher zBMI values were also associated with having a history of asthma and diabetes, consistent with studies from the U.S. and the U.K.[31,32] These associations between demographic characteristics and zBMI were apparent in both HKCC and non-HKCC communities. Despite statistically significant differences in most demographic characteristics, and the associations between sociodemographic characteristics and zBMI, there were no significant differences in zBMI between HKCC and non-HKCC communities. This is likely due to their vastly similar demographic profile.

This study fills a gap in the literature by leveraging an EMR system (EMRALD) in the (pre-) evaluation of a large-scale, community-based intervention. The strengths of using EMRALD include a large sample of young children, many who are rostered to their family physician and are expected to have multiple visits with anthropometric measures recorded in their EMR over time. We demonstrated from comparison of baseline characteristics that EMRALD children are younger, more rural, with lower rates of immigrant households and less Chinese or South Asian compared to all children in Ontario. These differences may have some implications for the generalizability of results. However, the rate of overweight and obesity in the EMRALD cohort were similar to national averages, which is an important similarity. The availability of repeated measures of valid zBMI values on the same individual, collected throughout the HKCC program life cycle (i.e. prior to, during, and after program implementation) makes the future evaluation of the impact of the HKCC program on healthy weights feasible using EMRALD.

The analysis of child weights represents an important component of a more comprehensive evaluation.[5] Programs like the HKCC can have an impact both directly and indirectly on child healthy weights. Thus, it is critical to also evaluate changes in distal outcomes such as family- and community-level outcomes. Combining a broad set of outcomes with a process evaluation is necessary for a comprehensive understanding of the HKCC’s impact at the provincial level.

This study highlights the feasibility of using primary care EMR data for community-level healthy weights evaluations, particularly when funded evaluations that collect objectively measured heights and weights are not feasible. Policy makers should support additional primary care practices to make their EMR data available, as this would further improve the generalizability of EMR-based databases available for research and evaluation. Currently, EMR data is contributed voluntarily by physicians and there is no mandatory reporting of any data elements. Further, the use of primary care EMR data for public health purposes, such as evaluation and surveillance, could be a point of collaboration between public health and primary care. Monitoring growth in childhood is critical to understanding the health of individuals and the community; common goals for both public health and primary care.

Our study does have some limitations. First, as previously reported in the literature,[6] the use of standardized equipment to measure height and weight by family physicians providing data to EMRALD is unknown, which might affect the accuracy of these measurements. However, one recent survey of primary care providers in Ontario found the use of recommended equipment for weight and height measurements was high; although the use of recommended length boards in children less than 2 years was low.[33] Although we excluded around 1% of children from the cohort because their only zBMI measurement recorded during the observation period was biologically implausible, measurement and/or data entry errors may still exist in the data. To whatever extent these errors exist in the data, we would expect that they would be independent of HKCC community type and thus only bias the findings towards the null.

Second, the PCCF version used to define the HKCC communities was from 2013. However, availability of census data required that we use the 2006 PCCF to determine neighbourhood income quintile and rural residency. This difference may cause some misclassification for children with postal codes that changed census geographic characteristics, for example a community that was previously classified as rural (i.e. population <10 000 persons) may have experienced population growth over time. In addition, for children with newer postal codes, they would be classified as neighbourhood income quintile missing or rural residence unknown. Third, our classification of ethnicity was limited to an algorithm that identifies individuals of Chinese and South Asian ethnicity. Ethnicity information is not routinely collected in Ontario, which means that our analyses had a limited ability to detect or control for differences in zBMI based on ethnicity. Last, results involving the immigration status characteristic should be interpreted with caution. We were only able to determine whether the child (or their linked-birth mother) had a documented landing date before January 1, 2013 because of Immigration, Refugees and Citizenship Canada (IRCC) data availability at the time of analysis. Thus children in the cohort who were recent immigrants would have been misclassified. In addition, we were unable to determine the immigration status of any other member of the child’s household. To whatever extent that the aforementioned limitations impacted our sample, we would expect them to do so independent of community type and thus not significantly bias the main findings regarding prevalence of high zBMI.

In conclusion, we found that prior to the HKCC program launch, zBMI of children aged 1–12 years were similar inside and outside of HKCC community boundaries. While there are limits to EMRALD, the linkage between electronic medical records and administrative databases provides a rich data source that is valuable for surveillance and evaluation. This report supports the feasibility of using EMRALD to evaluate community-level interventions like the HKCC program.

## Supporting information

S1 Table Proportion of children in body mass index z-score (zBMI) categories overall and by HKCC community status, by sex, ages 1–12. (DOCX)

S2 Table Proportion of children in body mass index z-score (zBMI) categories overall and by HKCC community status, by age group. (DOCX)
