# Supplementary material for: Using EMRALD to assess baseline body mass index among children living within and outside communities participating in the Ontario, Canada Healthy Kids Community Challenge
Source: PLoS One. 2019 Apr 11;14(4):e0213443. doi: 10.1371/journal.pone.0213443 (PMC6459483; doi:10.1371/journal.pone.0213443)
Supplement: S1 Table — (DOCX) [file pone.0213443.s001.docx]

**S1 Table**. Proportion of children in body mass index z-score (zBMI) categories overall and by HKCC community status, by sex, ages 1-12.

| Females | | | | | | | |
| --- | --- | --- | --- | --- | --- | --- | --- |
|  | HKCC Communities | | Non-HKCC Communities | |  | Total | |
|  | N | % (95% CI) | N | % (95% CI) | p-value | N | % (95% CI) |
| ≤-2 | 67 | 1.9 (1.5-2.4) | 128 | 2.1 (1.8-2.5) | 0.47 | 195 | 2.0 (1.8-2.3) |
| >-2 to ≤1 | 2 625 | 74.0 (72.6-75.5) | 4 433 | 72.8 (71.7-73.9) | 0.19 | 7 058 | 73.3 (72.4-74.1) |
| >1 to ≤2 | 615 | 17.3 (16.1-18.6) | 1 056 | 17.4 (16.4-18.3) | 1.00 | 1 671 | 17.3 (16.6-18.1) |
| >2 to ≤3 | 193 | 5.4 (4.7-6.2) | 394 | 6.5 (5.9-7.1) | 0.04 | 587 | 6.1 (5.6-6.6) |
| >3 | 46 | 1.3 (1.0-1.7) | 77 | 1.3 (1.0-1.6) | 0.89 | 123 | 1.3 (1.1-1.5) |
| Males | | | | | | | |
|  | HKCC Communities | | Non-HKCC Communities | |  | Total | |
|  | N | % (95% CI) | N | % (95% CI) | p-value | N | % (95% CI) |
| ≤-2 | 85 | 2.2 (1.8-2.7) | 115 | 1.8 (1.5-2.1) | 0.12 | 200 | 1.9 (1.7-2.2) |
| >-2 to ≤1 | 2 717 | 70.8 (69.4-72.3) | 4 513 | 70.0 (68.8-71.1) | 0.36 | 7 230 | 70.3 (69.4-71.2) |
| >1 to ≤2 | 669 | 17.4 (16.3-18.7) | 1 199 | 18.6 (17.7-19.6) | 0.14 | 1 868 | 18.2 (17.4-18.9) |
| >2 to ≤3 | 273 | 7.1 (6.3-8.0) | 458 | 7.1 (6.5-7.8) | 0.98 | 731 | 7.1 (6.6-7.6) |
| >3 | 92 | 2.4 (1.9-2.9) | 165 | 2.6 (2.2-3.0) | 0.62 | 257 | 2.5 (2.2-2.8) |
