# Supplementary material for: Using EMRALD to assess baseline body mass index among children living within and outside communities participating in the Ontario, Canada Healthy Kids Community Challenge
Source: PLoS One. 2019 Apr 11;14(4):e0213443. doi: 10.1371/journal.pone.0213443 (PMC6459483; doi:10.1371/journal.pone.0213443)
Supplement: S2 Table — (DOCX) [file pone.0213443.s002.docx]

**S2 Table**. Proportion of children in body mass index z-score (zBMI) categories overall and by HKCC community status, by age group.

| **Ages 1-3** | | | | | | | |
| --- | --- | --- | --- | --- | --- | --- | --- |
|  | **HKCC Communities** | | **Non-HKCC Communities** | |  | **Total** | |
|  | **N** | **% (95% CI)** | **N** | **% (95% CI)** | **p-value** | **N** | **% (95% CI)** |
| **≤-2** | 61 | 2.3 (1.8-2.9) | 84 | 2.0 (1.6-2.5) | 0.47 | 145 | 2.1 (1.8-2.5) |
| **>-2 to ≤1** | 1 929 | 72.6 (70.8-74.3) | 2 906 | 70.4 (68.9-71.7) | 0.048* | 4 835 | 71.2 (70.1-72.3) |
| **>1 to ≤2** | 505 | 19.0 (17.5-20.5) | 840 | 20.3 (19.1-21.6) | 0.18 | 1 345 | 19.8 (18.9-20.8) |
| **>2 to ≤3** | 132 | 5.0 (4.2-5.9) | 258 | 6.3 (5.5-7.0) | 0.03* | 390 | 5.7 (5.2-6.3) |
| **>3** | 31 | 1.2 (0.8-1.7) | 43 | 1.0 (0.8-1.4) | 0.63 | 74 | 1.1 (0.9-1.4) |
| **Ages 4-8** | | | | | | | |
|  | **HKCC Communities** | | **Non-HKCC Communities** | |  | **Total** | |
|  | **N** | **% (95% CI)** | **N** | **% (95% CI)** | **p-value** | **N** | **% (95% CI)** |
| **≤-2** | 69 | 2.1 (1.6-2.6) | 87 | 1.6 (1.3-1.9) | 0.09 | 156 | 1.8 (1.5-2.0) |
| **>-2 to ≤1** | 2 558 | 76.1 (74.6-77.5) | 4 220 | 76.0 (74.8-77.1) | 0.87 | 6 778 | 76.0 (75.1-76.9) |
| **>1 to ≤2** | 500 | 14.5 (13.7-16.1) | 860 | 15.5 (14.5-16.5) | 0.44 | 1 360 | 15.3 (14.5-16.0) |
| **>2 to ≤3** | 166 | 4.9 (4.2-5.7) | 281 | 5.1 (4.5-5.7) | 0.80 | 447 | 5.0 (4.6-5.5) |
| **>3** | 68 | 2.0 (1.6-2.6) | 108 | 1.9 (1.6-2.3) | 0.79 | 176 | 2.0 (1.7-2.3) |
| **Ages 9-12** | | | | | | | |
|  | **HKCC Communities** | | **Non-HKCC Communities** | |  | **Total** | |
|  | **N** | **% (95% CI)** | **N** | **% (95% CI)** | **p-value** | **N** | **% (95% CI)** |
| **≤-2** | 22 | 1.6 (1.0-2.4) | 72 | 2.5 (2.0-3.2) | 0.06 | 94 | 2.2 (1.8-2.3) |
| **>-2 to ≤1** | 855 | 62.7 (60.1-65.3) | 1 820 | 63.8 (62.0-65.6) | 0.49 | 2 675 | 63.5 (62.0-64.9) |
| **>1 to ≤2** | 279 | 20.5 (18.4-22.7) | 555 | 19.5 (18.0-21.0) | 0.45 | 834 | 19.8 (18.6-21.0) |
| **>2 to ≤3** | 168 | 12.3 (10.6-14.2) | 313 | 11.0 (9.9-12.2) | 0.20 | 481 | 11.4 (10.5-12.4) |
| **>3** | 39 | 2.9 (2.0-3.9) | 91 | 3.2 (2.6-3.9) | 0.56 | 130 | 3.1 (2.6-3.7) |

*statistically significant difference p-value <0.05
